# Integration of clinical perspective into biomimetic bioreactor design for orthopedics

**DOI:** 10.1002/jbm.b.34929

**Published:** 2021-09-12

**Authors:** Victoria Drapal, Jordan M. Gamble, Jennifer L. Robinson, Candan Tamerler, Paul M. Arnold, Elizabeth A. Friis

**Affiliations:** ^1^ Bioengineering Program University of Kansas Lawrence Kansas USA; ^2^ Department of Mechanical Engineering University of Kansas Lawrence Kansas USA; ^3^ Department of Chemical and Petroleum Engineering University of Kansas Lawrence Kansas USA; ^4^ Institute for Bioengineering Research University of Kansas Lawrence Kansas USA; ^5^ Carle School of Medicine University of Illinois‐Champaign Urbana Champaign Illinois USA

**Keywords:** biomimetic, bioreactor, bone, cartilage, clinical relevancy, multiple stimuli, orthopedics

## Abstract

The challenges to accommodate multiple tissue formation metrics in conventional bioreactors have resulted in an increased interest to explore novel bioreactor designs. Bioreactors allow researchers to isolate variables in controlled environments to quantify cell response. While current bioreactor designs can effectively provide either mechanical, electrical, or chemical stimuli to the controlled environment, these systems lack the ability to combine all these stimuli simultaneously to better recapitulate the physiological environment. Introducing a dynamic and systematic combination of biomimetic stimuli bioreactor systems could tremendously enhance its clinical relevance in research. Thus, cues from different tissue responses should be studied collectively and included in the design of a biomimetic bioreactor platform. This review begins by providing a summary on the progression of bioreactors from simple to complex designs, focusing on the major advances in bioreactor technology and the approaches employed to better simulate *in vivo* conditions. The current state of bioreactors in terms of their clinical relevance is also analyzed. Finally, this review provides a comprehensive overview of individual biophysical stimuli and their role in establishing a biomimetic microenvironment for tissue engineering. To date, the most advanced bioreactor designs only incorporate one or two stimuli. Thus, the cell response measured is likely unrelated to the actual clinical performance. Integrating clinically relevant stimuli in bioreactor designs to study cell response can further advance the understanding of physical phenomenon naturally occurring in the body. In the future, the clinically informed biomimetic bioreactor could yield more efficiently translatable results for improved patient care.

## 1 INTRODUCTION TO CLINICALLY RELEVANT BIOREACTORS

A bioreactor, put simply, is a vessel that maintains a specific microenvironment and allows biochemical reactions to occur.^1^ In tissue engineering (TE) applications, the microenvironment must be closely monitored and tightly controlled to ensure a high degree of accuracy and reproducibility amongst biological constructs.^2^, ^3^ These properties have made bioreactors an indispensable component of any bioprocess, irrespective of the end product, which can take the form of chemicals, pharmaceuticals, cells, tissues, or organs. Early bioreactors focused mainly on controlling purely environmental factors, such as temperature, pH, aeration, agitation, pressure, nutrient concentration, and waste removal.^2^, ^4^, ^5^ However, in certain fields, such as TE, a new era of bioreactors has arisen to incorporate additional physiologically‐derived factors, such as mechanical, electrical, and chemical cues.^1^ Despite these major changes in design, the common thread connecting all bioreactors is the ability to provide a controlled microenvironment for a product of interest. In TE and other research applications, the purpose of controllability is to evoke the scientific method; to be able to isolate variables and measure changes in response to variation, allowing for easier automation and reproducibility of experiments, which is essential for any study design.

**FIGURE 1:**
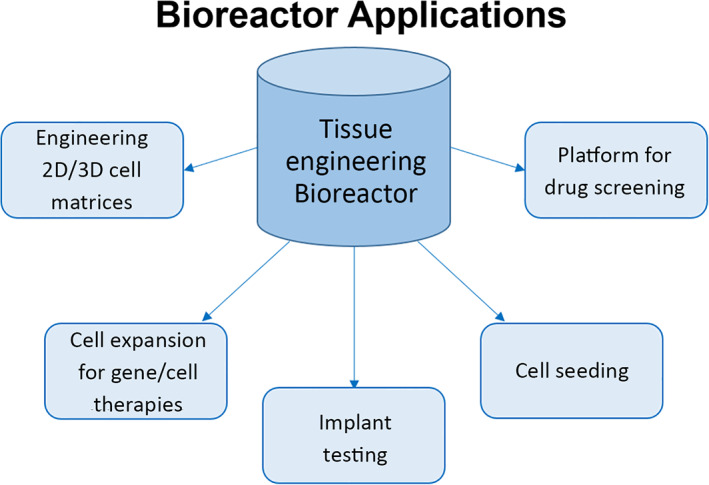
Various applications for TE bioreactors

Classical TE techniques typically involve the seeding of cells onto a supporting matrix, or scaffold, and supplying additional growth factors to promote cell adhesion, alignment, migration, proliferation, differentiation, and new tissue production.^6^ This combination of cells, scaffolding, and growth factors is known as the TE paradigm.^6^, ^7^, ^8^ Bioreactors are often added to the triad to supply biophysical stimulation to the cell scaffold and improve tissue formation metrics. Bioreactors in TE have three main uses: to maintain a specific cellular microenvironment, whether that be physiological or pathological, in order to better understand cell and molecular physiology/pathophysiology; to expand cell lines for gene/cell therapies or grow functioning tissue *in vitro* for clinical applications; and to test potential treatments for new therapeutic targets.^7^, ^9^ Bioreactors also have other clinical uses, such as testing biomedical implants and facilitating cell seeding onto scaffolds (Figure 1). For many pharmaceuticals and implants, pre‐clinical animal models are required by the FDA for testing. Bioreactors have the potential to replace pre‐clinical animal testing models, saving labor, time, and money.^10^

Research oriented bioreactors have had variable success over the years in the cultivation of engineered tissues, partly due to the lack of standardized environmental parameters and loading regimes.^11^ Yet despite the research success seen for specific bioreactor configurations, translation to the clinical setting has been limited. Classical TE methods have had fairly limited success for growing tissues *in vitro* and are only consistently used for thin or avascular tissues, such as skin, due to the diffusion limit of oxygen (100–200 μm).^10^, ^12^ As for larger, vascular tissues, like those needed to treat large bone defects, the speed of vascularization after implantation is often too slow to ensure adequate nutrient transport to the core.^12^, ^13^ Thus, *in vitro* strategies for enhancing vascularization before implantation are being investigated. Biomimetic bioreactors are being explored as one of these potential strategies, since research has implied that a well‐established, physiologically relevant microenvironment helps promote tissue growth and vascularization *in vitro*.^10^, ^13^, ^14^, ^15^ If these barriers can be addressed, the gap between research and clinical bioreactors will narrow and clinical translation may be feasible for improved patient care.

To better recapitulate the *in vivo* microenvironment, modern bioreactor designs for TE applications have shifted away from classical systems, which only focus on environmental cues and nutrient transfer, toward more specialized systems that incorporate additional biophysical stimuli, such as compressive loading^16^, ^17^ or electrical polarization,^18^, ^19^ to facilitate specific cell behaviors. These physiological stimuli are especially relevant for bone tissue engineering (BTE) applications where mechanical and electrical loads play a key role in cell signaling and long‐term tissue functionality.^3^

Several groups have designed modern bioreactors that strive toward enabling long‐term *in vitro* tissue functionality. However, these designs typically involve only a single biophysical stimulation type. For example, some bioreactors use purely mechanotransduction principles for musculoskeletal tissues,^11^, ^16^, ^17^, ^20^, ^21^, ^22^, ^23^, ^24^, ^25^ for example, Schreivogel et al. where they investigated the effects of cyclic mechanical compression of human bone marrow derived stromal cells (hBMSCs),^26^ while other bioreactors focus only on the influence of electrical stimuli,^15^, ^27^, ^28^, ^29^, ^30^, ^31^, ^32^, ^33^, ^34^, ^35^ for example, Gittens et al. introduced electrical stimulation to osteoblasts through titanium substrates and showed an increase in osteoblast differentiation.^18^ Mechanical loading regimes have been a particular topic of interest in promoting *in vitro* cell expansion due to the increasingly available information on the role of mechanotransduction in cell signaling pathways.^3^, ^36^, ^37^ Various mechanical stimulation techniques have been used to improve growth, maturation, and function in several engineered tissues, such as bone,^16^, ^17^, ^37^, ^38^ cartilage,^39^, ^40^ smooth muscle,^41^ and tendons.^42^ Despite the individual successes found in these studies, emerging evidence from research into the various cellular signal transduction pathways suggests that multiple integrated stimuli could further enhance tissue growth and functionality.^43^, ^44^, ^45^

**FIGURE 2:**
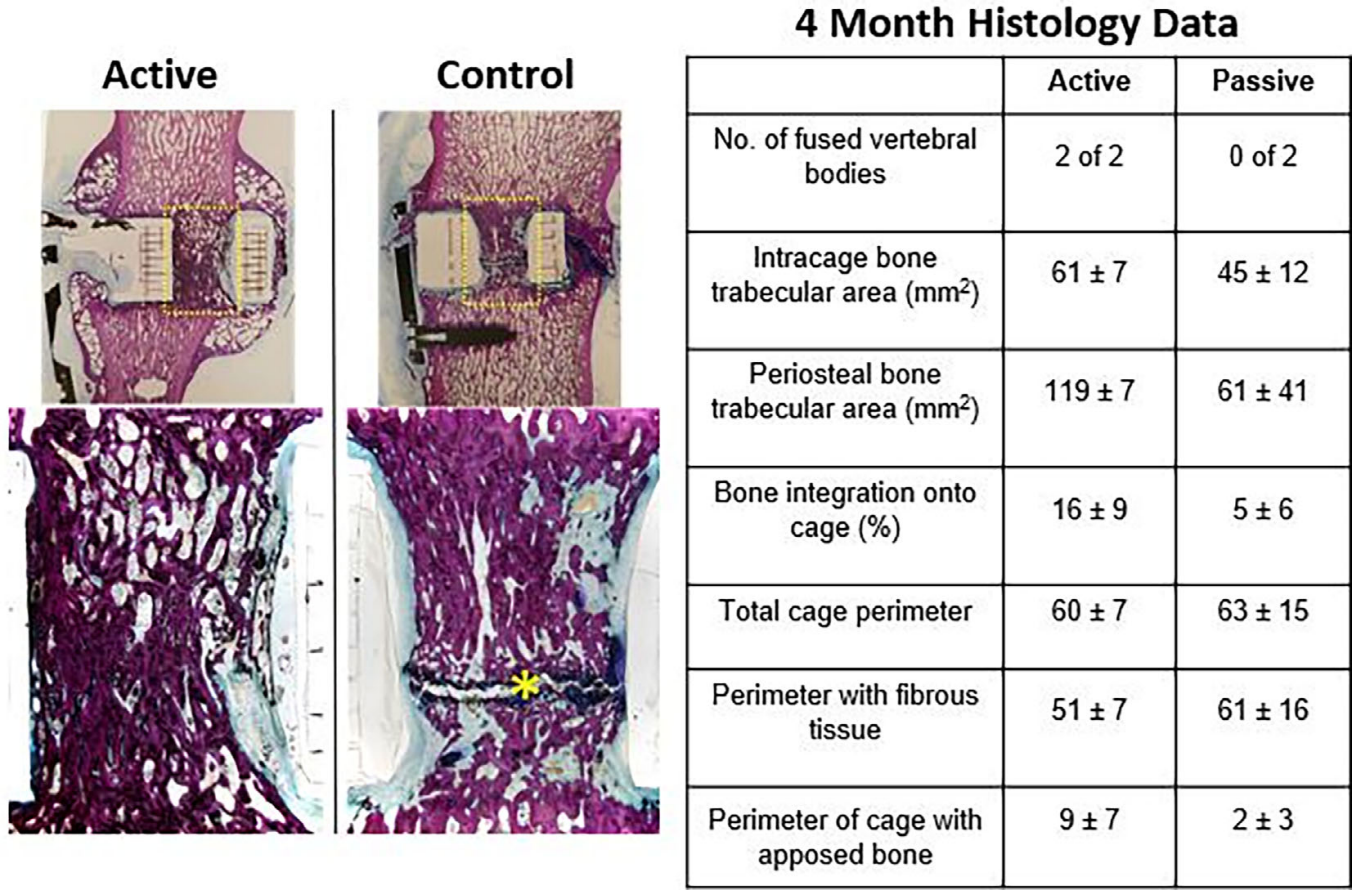
Histological data after 4 months of electrical stimulation with a spinal implant device in the lumbar spines of ovine models. Adapted from Friis et al^46^

In a pilot ovine study conducted by Friis and Arnold, a novel piezoelectric interbody implant was placed in the lumbar segment of the spine to assess fusion progression and bone growth, while two other levels of the spine served as controls.^46^ The circuitry accompanying the piezoelectric materials inside the implant produced a capped negative current on the electrode of the implant surface in sync with sufficient mechanical loading.^46^ This form of mechanically synced electrical stimulation (MSES) is inherently distinct from the presently available battery‐operated implantable electrical stimulation devices that produce constant direct current irrespective of the mechanical load.^46^ The ovine study included radiographic fusion assessment at 6 weeks and 4 months, biomechanical testing, and histological analysis at 4 months, to assess fusion mass and time‐to‐fusion (Figure 2).^46^ The results of this pilot ovine study showed that spine segments treated by MSES with the piezoelectric device had an earlier and more robust fusion, as measured radiographically, biomechanically, and histologically.^46^ Additionally, no pathologic bone formation or adverse bone growth were detected in the spinal canal.^46^ The piezoelectric implant supplied a coupled mechanical and electrical load to the bone resulting in faster fusion, which supports the theory that multiple integrated stimuli could enhance bone tissue formation. However, the cellular mechanism for this enhanced bone growth remains unknown, and thus exemplifies a need for a biomimetic bioreactor that utilizes multiple stimuli to mimic the *in vivo* environment and provide quantitative analysis.

This review article stresses the need for clinically informed, multiple stimuli, TE bioreactors by first providing a brief historical summary on the progression of bioreactors, from classical to modern, describing the major advances in design with respect to clinical significance. Next, a comprehensive overview of the individual biophysical stimuli and their role in establishing a biomimetic microenvironment for TE, with a specific focus on the cellular response to specific loading regimes is provided. Finally, a model for future TE bioreactors that simultaneously incorporates several of these biophysical stimuli to create a more physiologically relevant microenvironment for specific cell types is also presented. Even the most advanced bioreactor models cannot recapitulate a physiological environment accurately. Overall, the need to integrate diverse stimuli simultaneously to drive cells toward a desired phenotype and offer a clinically informed bioreactor design for a patient‐first mindset for diagnosis and treatment is highlighted.

## 2 EVOLUTION OF TISSUE ENGINEERING BIOREACTORS

**FIGURE 3:**
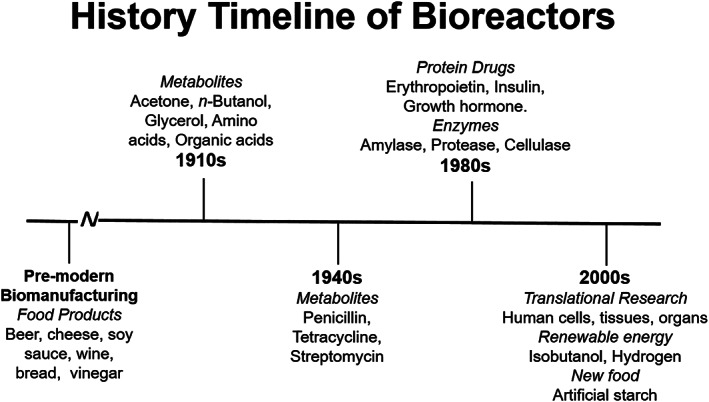
Bioreactors throughout time with key products represented. Adapted from Zhang, YP, Sun J, Ma Y. Biomanufacturing: history and perspective. *J Ind Microbiol Biotechnol*. 2017;44(4,5):773‐784

One of the earliest instances of industrial fermenters, before the term bioreactor was even coined, was developed by Chaim Weizmann in the early twentieth century to produce butanol and acetone on a large‐scale for the war effort.^5^, ^47^ Since then, bioreactors have been constantly adapting to meet the needs of the end product (Figure 3). With the increasing demand for mammalian cell culture production came the need for two more distinctive bioreactor applications: stimulating cell expansion/aggregation and promoting tissue formation. However, when culturing cells, static culture vessels, often used in early fermenters, do not provide adequate homogenous nutrient supplies.^48^ Different modes of operation have been employed in TE bioreactors with varying success and are classified by the nutrient input, waste output, and cell harvesting techniques.^49^ The mode of operation forms the core for each bioreactor design, since many engineering parameters are dictated by the mass transfer, aeration, and nutritional needs.

### 2.1 Modes of operation

**FIGURE 4:**
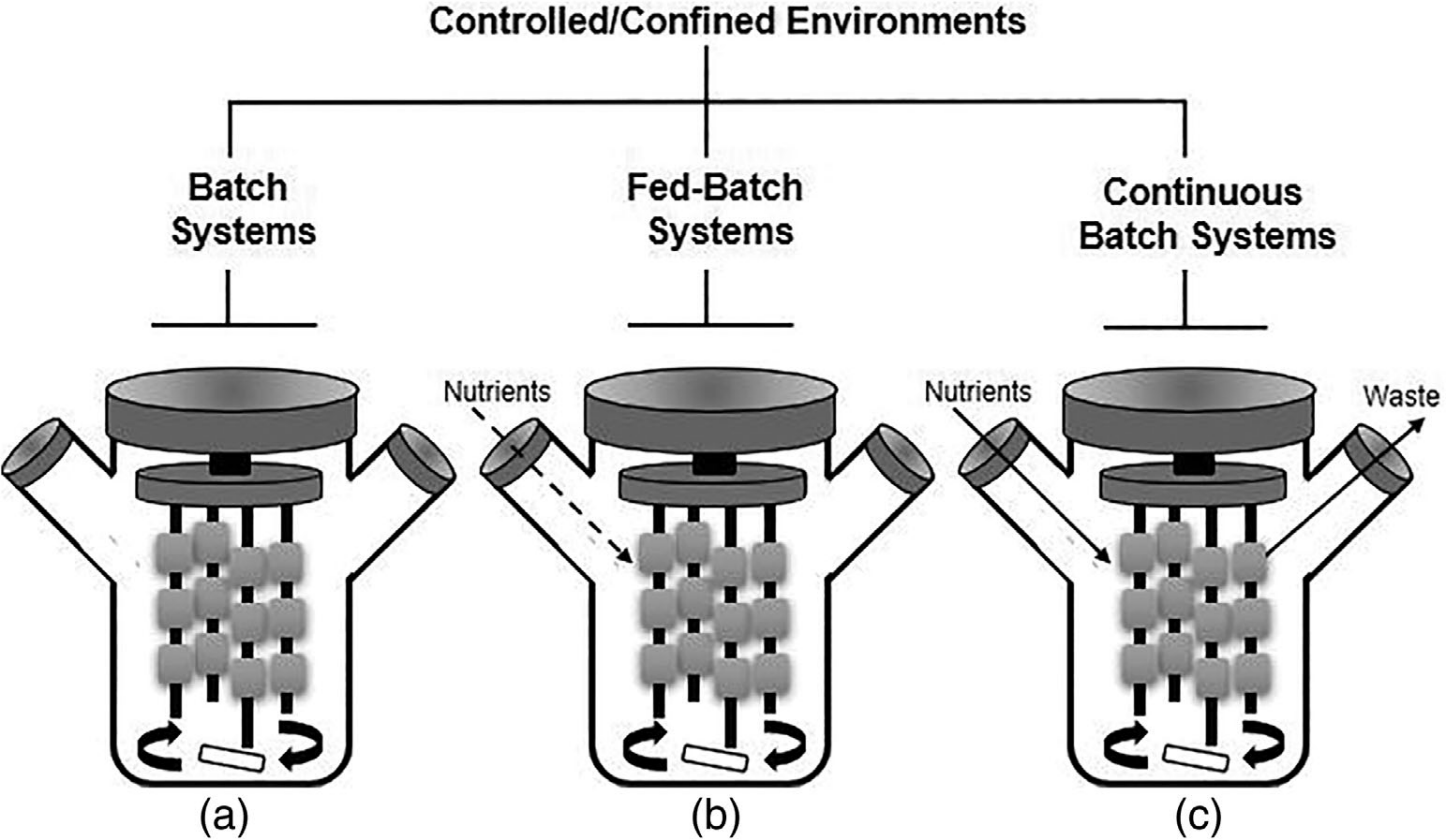
A schematic depicting the differences and similarities between batch, fed‐batch, and continuous batch systems in a spinner flask. (a) Batch system where media is introduced a singular time. (b) Fed‐batch system where nutrients are added more than one time. (c) Continuous batch system where a constant perfusion of nutrients is introduced, and waste is eliminated

Among the various modes of operation available for bioreactors used in submerged liquid cultures, three primary modes of operation include batch, fed‐batch, and continuous batch systems (Figure 4).^49^ Understanding these main modes of operation is important to help determine the most suitable one for a given application. Batch operation, which is the simplest mode, represents a closed system where all the nutrients required for growth are provided at the beginning of the process (Figure 4a). Fed‐batch begins as a batch process, then changes the operation to an open system by adding nutrients and/or inducers through an inlet (Figure 4b). Finally, in continuous batch operation, the system environment is maintained without any fluctuations in nutrient quantity, cell numbers, or cell mass, simply operating at steady state (Figure 4c). In general, any bioreactor system can be adapted to run any of the modes depending on the environmental requirements. However, certain modes may be more effective for TE applications.^49^ For example, continuous batch systems allow for the development of a more physiological relevant environment for bone. Tissue compartments that regularly get flushed with dynamic fluids, such as blood, interstitial fluid, or synovial fluid exchange, experience similar flow patterns with those found in continuous batch systems.^50^, ^51^ With proper optimization, correct levels of oxygen influx, nutrient consumption, and waste removal allows for the biomimicry needed for clinically relevant work.

### 2.2 Classical tissue engineering bioreactors

**FIGURE 5:**
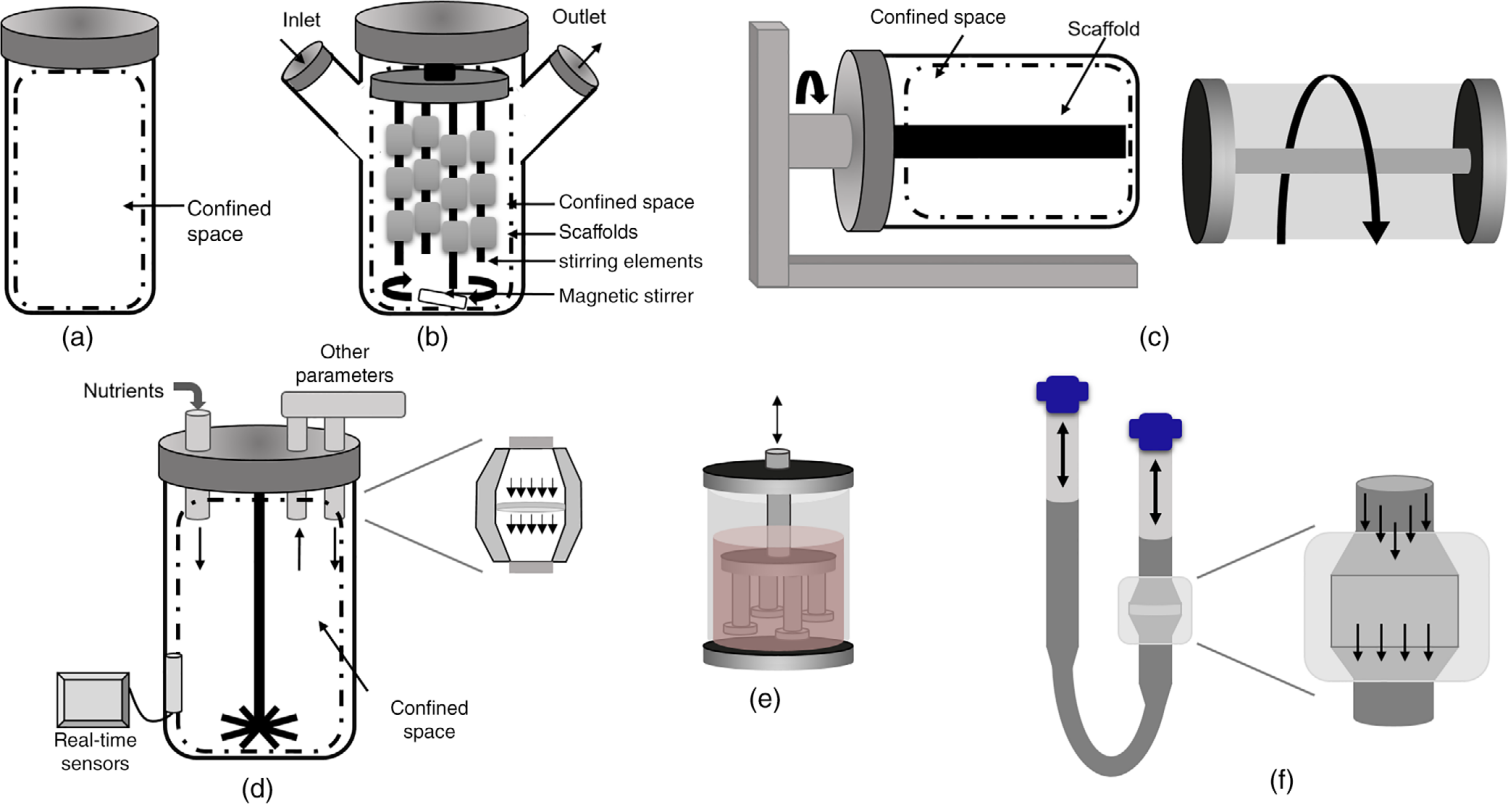
Schematic of the various types of bioreactors: (a) static culture, (b) spinner flasks, (c) rotating wall vessels, (d) perfusion flow bioreactors, (e) compression bioreactors, and (f) tubular flow. Adapted from Chen C, Hu Y. Bioreactors for tissue engineering. *Biotechnol Lett* 2006;28:1415‐1423

TE bioreactors can historically be split into either classical or modern categories. Classical bioreactor systems control several environmental parameters, use a continuous mode of operation for homogeneous nutrient supply, and use an agitation method. Modern bioreactor systems build off the classical system by incorporating an additional mechanical, electrical, or chemical stimulation type. Before research surfaced suggesting that external stimulation, such as compressive or electrical loading, could influence cellular behavior,^52^, ^53^ early bioreactor designs focused on media mixing techniques to optimize nutrient transfer and inherently apply small amounts of shear stresses to cells. These types of classical bioreactors can range from simple to more complicated mixing techniques and include the following: stirred‐tank, spinner flask, rotating wall vessel, and perfusion flow bioreactors (Figure 5).^54^, ^55^, ^56^

One of the most conventional bioreactors in chemical engineering and biopharmaceuticals is the stirred‐tank bioreactor.^54^ The main characteristics of stirred‐tank bioreactors are the free‐floating cells in media and the agitator arm (or impeller). The impeller performs a wide array of functions, including heat and mass transfer, aeration, and mixing.^54^ The geometric parameters of the impeller and tank, such as the off‐bottom clearance, the impeller size, and the ratio of liquid height to tank diameter, can affect the performance of the stirred‐tank bioreactor.^54^ Typically, stirred‐tank bioreactors produce high shear stresses, which are not ideal for fragile mammalian cell cultures. Thus, for TE applications, stirred‐tank bioreactor designs are modified to reduce damage to cells by minimizing hydrodynamic shear forces induced by agitation and air bubbles.^57^, ^58^

Spinner flasks are very similar to stirred‐tank bioreactors, except the cells are seeded on either scaffolds, that are fixed to needles protruding from the vessel, or onto microcarriers.^55^, ^59^ The stirring element also differs from the typical stirred‐tank bioreactor. Frith et al. describe spinner flasks as comprised of a magnetic stirring arm with two side arms that allow for the addition or subtraction of substance and aeration to the contents of the flask.^60^ The side‐arms, also referred to as the inlet and the outlet to the confined space, contain filters that help limit contamination while also ensuring oxygenation. Spinner flasks are typically used to expand embryonic and adult stem cells (e.g., human hematopoietic stem and progenitor cells, human embryonic stem cells, and mouse embryonic stem cells) for both research and stem cell therapy purposes.^48^ Spinner flasks are also routinely used for dynamic cell seeding^61^ and inducing specific geometries for cell clusters.^62^ Despite the success and versatility of spinner flasks, some drawbacks include the possible formation of a dense superficial cell layer, which could block oxygen and nutrient supplies to the construct's core, and the formation of shear stress gradients from nonhomogeneous forces.^48^, ^55^ Spinner flasks are also routinely used for dynamic cell seeding^61^ and inducing specific geometries for cell clusters.^62^

Rotating wall vessels (RWV), or rotary cell culture systems (RCCS), were first used to simulate microgravity conditions, but have since been adapted for use in dynamic three‐dimensional (3D) culture systems.^55^ Frith et al. describes rotating wall vessels as cylindrical chambers with internal, membrane‐covered cylinders that aid in drawing oxygen into the space while rotating the vessel.^60^ The rotating wall helps to diffuse nutrients in the media by generating high mass transfer rates and small levels of shear stress. The cells can be rigidly attached to the wall via microcarrier scaffolds, rigidly attached to the core via rotating beds, or free‐falling.^55^ For the free‐falling environment, the cells are supported by balancing the forces acting on the construct. The applied forces are the drag force (*F*_d_), the centrifugal force (*F*_c_), and the gravitational force (*F*_g_).^59^ The rotation of the wall around the scaffold creates an equilibrium of forces that allows for the scaffold to be held up in the medium without colliding with the vessel. Successful growth of human hematopoietic stem and progenitor cells,^63^ human mesenchymal stem cells (hMSCs),^64^ and mouse embryonic stem cells have been shown in RCCS.^65^ Changes in cell behavior have also been identified between cells cultured in RWVs and spinner flasks, suggesting that the dynamic 3D environment influences MSC properties.^60^ Although RWVs have been demonstrated to promote differentiation, some controversy exists on whether the dynamic environment promotes or inhibits osteogenesis. For example, some studies have shown that RWVs increase adipogenesis but decrease osteogenesis,^66^ while others present evidence that RWVs increase both.^60^

Perfusion flow bioreactors are the most commonly used bioreactor for TE applications involving three‐dimensional bone stimulation due to overcoming the diffusional limitations of rotation‐based and stirring bioreactors.^50^ Perfusion flow reactors are commonly used for seeding scaffolds and culturing the subsequent construct. Scaffold seeding with perfusion bioreactors produces a more uniform cell distribution compared with other agitation methods.^2^ In perfusion bioreactors, solutions under continuous or pulsatile laminar flow are pumped through the entire scaffold, enabling mass transport of nutrients and oxygen.^55^ The fluid flow produced by perfusion bioreactors also exhibit flow patterns most similar to those experienced in native tissues, such as bone and blood vessels. Perfusion bioreactors can be further divided into systems using direct or indirect media flow. In direct perfusion, the construct is tightly sealed, forcing the flow to travel through the construct's pores. However, in indirect perfusion there is only a loose seal, allowing for the flow to take the path of least resistance.^55^ Since direct perfusion allows for easier control of flow‐induced stresses on the construct, it is often preferred to indirect perfusion. MSCs are one of the most common cell types used in perfusion bioreactors.^48^ There have been reports that lower perfusion rates increase MSC expansion, while higher perfusion rates decrease expansion due to the effects of high hydrodynamic stress.^48^ Thus, careful design considerations must be given to ensure the hydrodynamic stresses produced by the fluid flow do not damage the cells. In one study, a perfusion‐based microbioreactor for rapid cellular disease diagnosis was designed with piezoelectric transducer integration in order to closely measure the shear stresses being applied to the cells.^67^ The microbioreactor design was modified in order to maintain shear stresses in the milli‐Pascal range while still applying fluid volume flow rates between 0.03 and 3 μL/min.^67^

While all the classical bioreactors mentioned in this section are still used in the field of TE today, there has been a shift toward perfusion bioreactors due to the higher control of shear stresses and uniform flow. It can be argued that a perfusion flow system coupled with a continuous batch mode creates an environment that best recapitulates the physiological environment for most biological tissues, and thus allows for better translation into the clinical realm. The driving factor for the shift from classical to modern bioreactors, and the driving factor behind all TE bioreactor designs, is the desire to improve tissue formation metrics by simulating the *in vivo* microenvironment. Future bioreactors should also be designed with these goals in mind.

## 3 BIOREACTOR ENVIRONMENTAL REQUIREMENTS

Recall that a TE bioreactor's main purpose is to create and maintain a specific microenvironment for biological constructs. This microenvironment is designed to elicit specific outcomes, such as a change in cell phenotype, maintaining cellular metabolism, or promoting cellular proliferation. It is important to note that the cellular outcome is not only dependent on the microenvironment but also the types of cells used in the biological construct. For example, a specific microenvironment might promote cellular maturation in one cell type but hinder the expression of another cell type's desired phenotype. Thus, it is important to choose and optimize the microenvironment toward a specific cell type and desired outcomes. Thus, when designing a bioreactor, understanding the factors that promote or inhibit specific cell functions is important to ensure that the appropriate microenvironment is being created.^1^

The microenvironment can be broken up into two types: the physiochemical environment and the physiological environment. The physicochemical environment embodies the abiotic factors of the system and involves conditions such as temperature, pH, pressure, O_2_ and CO_2_ levels, and ionic strength of the culture media.^68^ On the other hand, the physiological environment is less defined, but is generally known to accept all other forms of stimulation outside of those classified as physicochemical. In the early days of TE, the physiological environment included nutrient concentration, hormones, cell scaffolding, and mass transfer techniques. However, the physiological environment can also include direct cell loading, whether that be mechanical, electrical, or chemical (Table 1).^1^

**TABLE 1:** Examples of various stimuli and the factors associated with them

Stimulus	Factors
Environmental	Gases (O_2_, CO_2_)
	Temperature
	Pressure
	Fluid flow
	pH
Chemical	Cytokines
	Hormones
	Small molecules
	Nutrients (media)
	Growth factors
	Other cells
Mechanical	Compression
	Shear
	Torsion
	Tension
	Ultrasound
Electrical	Impedance
	Voltage
	Resistance

Optimizing the multifactorial microenvironmental cues and protocols for TE bioreactors requires a combinatorial approach to assess the effects of the integrated stimuli simultaneously.^69^ The environment created within the bioreactor must also allow enough nutrients and support for cellular growth. These factors can include nutrients within the media: controlling pH levels in the solution allowing oxygen flow throughout the chamber and temperature controls. Additional cues on top of these base chemical cues act as the independent variables in study designs.

Although it is important to keep optimizing multifactorial cues and advancing bioreactor technology in order to create a more accurate *in vivo* microenvironment, efforts also need to be focused on better understanding what the physiological microenvironment actually is for a given tissue type and localization. For example, it was not until Fukada and Yasuda in 1957 that bone was discovered to have piezoelectric properties, thus prompting research into the effects of electrical stimulation on bone regeneration.^52^ This discovery opened new avenues for researchers to tackle bone remodeling and TE bioreactor design. On the chemical side, there are constantly new interactions being discovered between key signaling molecules during the bone remodeling process.^43^, ^70^ It is essential for TE bioreactor designs to adapt every time new information about the physiological environment surfaces. Although it is unlikely that construction of a true *in vivo* environment can ever be made *in vitro*, the quality of tissue engineered constructs is sure to improve as researchers move closer to this goal.

## 4 BIOREACTOR STIMULATION TYPES

Single stimulus bioreactors for orthopedics attempt to promote the growth of bone and/or cartilage by creating a more physiologically accurate microenvironment compared with traditional TE techniques. Bone and cartilage undergo a variety of stimuli during everyday activities, such as compressive and tensile forces, high frequency vibrations, chemical signaling between cells, and even electrical signals produced by the brain. In order to simulate these microenvironments, single stimulus bioreactors have been designed, manufactured, and tested. Common capabilities of single stimulus bioreactors include compression for bone and cartilage,^11^, ^17^ incorporation of hormones such as the parathyroid hormone (PTH),^71^ and electrical stimulation for human embryonic cells,^72^ which have all shown more physiological results compared with static conditions.

### 4.1 Mechanical stimulation

Bone response to mechanical stimulation has long been a topic of interest. According to Wolff's law, bone is an adaptive tissue that changes its geometric and structural properties in order to meet the demands of its physical environment.^53^, ^73^ In other words, the mechanical loads applied to bone play an important role in determining its mechanical properties, such as the elastic modulus, ultimate tensile strength (UTS), hardness, and stiffness. Wolff's law can best be illustrated in disuse applications, or a significant reduction in motion and bone stimulation. Reduced mechanical loading, whether induced by injury, age, immobilization, or antigravity conditions, has been shown to lead to a significant decrease in bone density and strength.^74^ Wolff's law has been widely accepted as a simplified model of bone remodeling. However, it is still unknown how loads on a macroscale directly affect cells on a microscale. Current research in mechanical stimulation of tissue has mainly focused on these intracellular behaviors and the specific signal pathways that induce these responses.

**FIGURE 6:**
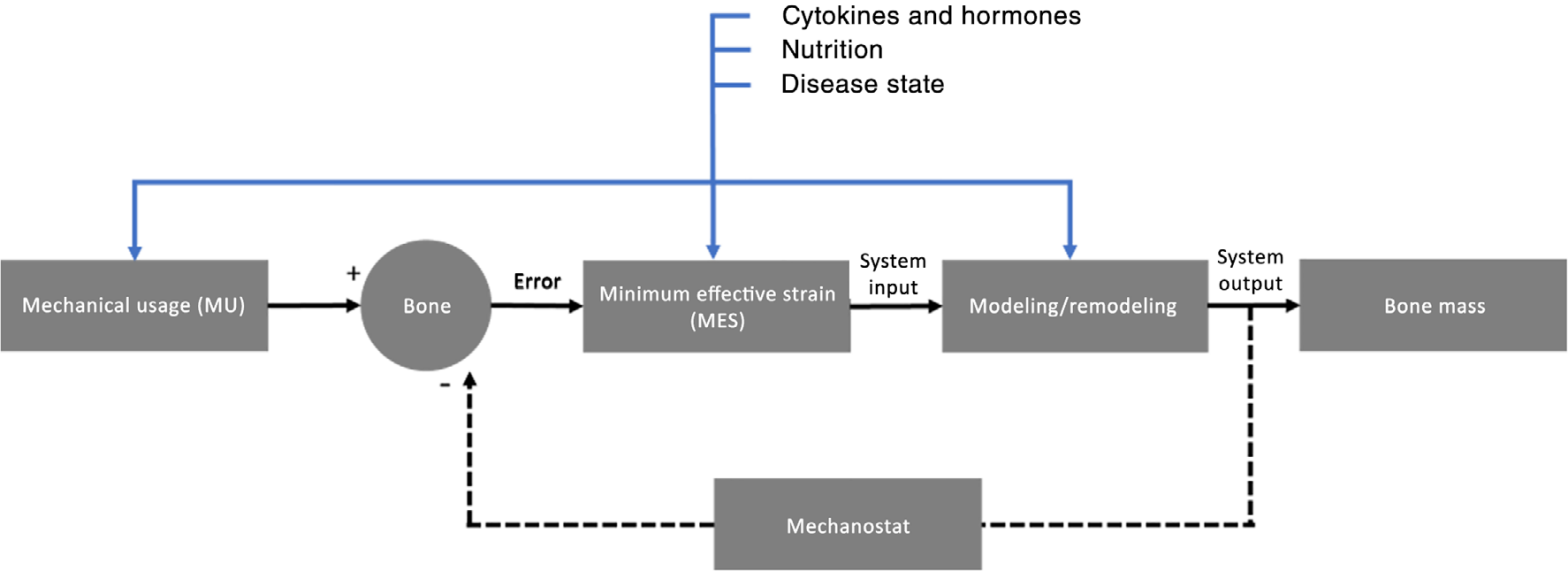
Flow diagram illustrating Frost's “mechanostat” theory. The model is represented as a simple feedback loop where bone is remodeled by sensing the change in bone mass and activating modeling/remodeling to better resist loads. Additional factors that can influence stages in the process are also included

In 1987, Frost built upon Wolff's law with his proposal of the Mechanostat Theory, which describes the influence of mechanical usage on bone structure by changing the bone's mass and architecture to resist habitual loads.^53^ Frost recognized that the mechanisms involved in bone adaptation mimic the behavior of a home thermostat, thus referring to it as a “mechanostat.”^53^ The theory models bone adaptation as a feedback control loop, where the mechanical usage is the reference point, the modeling/remodeling process is the plant, and the bone mass is the output (Figure 6). When the applied strain from mechanical usage falls above the minimum effective strain (MES), or the strain threshold needed to trigger bone modeling, then the bone adapts to better resist this strain. In bone, the MES is believed to be around 1,000–3,000 μstrain.^75^

Bone converts the strain exhibited from mechanical usage into electrochemical activity through a process known as mechanotransduction. These electrical and chemical signals can then activate a network of signal transduction pathways to promote or inhibit bone modeling/remodeling. Although the exact mechanisms of action remain unclear, the Wingless‐related integration site (Wnt) signaling pathway, and more specifically the inhibitor Sost/sclerostin, is suggested to play an important role in regulating bone adaptation.^76^ Furthering the understanding of the Wnt pathway may lead to the development of medical devices and therapeutic strategies directly targeting bone adaptation. The key cells believed to be responsible for sensing mechanical cues and regulating gene expression for bone adaptation, also known as the mechanostat component, are osteocytes.^77^ Thus, osteocytes, along with osteoblasts, osteoclasts, and their precursors, are generally the targeted cell types in mechanical bioreactors.

As with all biophysical stimuli, the loading regime plays a vital role on tissue and cell response, and since these regimes are not standardized, the success of mechanical bioreactors has been limited (Table 2). For example, Liu et al. looked at the cellular response of human bone mesenchymal stem cells (hBMSCs) seeded on scaffolds in response to two different cyclic compressive loading regimes incorporated in a perfusion bioreactor. *Loading Regime I* (10% strain, 0.5 Hz, 1 time/day, 8 hr/time, 16 hr of rest) showed significantly reduced cell proliferation, viability, protein concentration, and tensile modulus compared with *Loading Regime II* (10% strain, 0.5 Hz, 4 times/day, 2 hr/time, 4 hr of rest).^11^ This study exemplifies that the loading regime, specifically regarding the duration of the loading, plays a vital role in osteogenesis. Other important parameters of the loading regime include the strain magnitude, strain/flow rate, and frequency, each of which have shown to affect the biological output.^3^

**TABLE 2:** Summary of cellular behaviors in response to mechanical loading for *in vitro* and animal studies

Articles	Loading type	Loading regime	Loading duration	Cell/tissue type or animal model	Results	References
Maeda et al. (2017)	Compression	Compression: 1–2% strain, 3–4 cycles/min, 22 hr/day	3 days	Chick tibia explants	Viability ↓, elastic modulus ↑, ALP –	[17]
Liu et al. (2012)	Compression and perfusion	Perfusion rate of 10 ml/min. Compression I: 10% strain, 0.5 Hz, 1 time/day, 8 hr/time, with 16 hr of rest. Compression II: 10% strain, 0.5 Hz, 4 times/day, 2 hr/time with 4 hr of rest	14 days	hBMSC seeded scaffold	Proliferation ↑, viability ↑, equilibrium modulus ↑, tensile modulus ↑, procollagen I ↑	[11]
Birmingham et al. (2015)	Compression and perfusion	Perfusion rate 0.9 ml/min. Compression: ±0.3 g, 30 Hz, 1 hr/day	22 days	Cervical porcine vertebrae	Viability –, bone formation ↑, bone resorption ↑	[38]
Meinert et al. (2017)	Compression and shear	Compression: 30% strain, 1 Hz, 1 hr/day. Shear: 1 mm sliding amplitude, 1 Hz, 1 hr/day	28 days	Human articular cartilage seeded hydrogel	Collagen type II ↑, hyaline cartilage ECM deposition ↑	[79]
Kang et al. (2011)	Compression and ultrasound	Compression: 1.0 Hz, 10% strain, 20 min/day. Ultrasound: 1.0 MHz, 10 mW/cm^2^, 20 min/day	10 days	MC3T3‐E1 pre‐osteoblast seeded scaffold	Proliferation ‐, collagen type 1↑, osteocalcin ↑, RUNX2 ↑, osterix ↑	[20]
Huey and Athanasiou (2011)	Tension and compression	Tension and compression: 10% strain, 1 Hz, 1 hr/day	21 days	Human femoral articular cartilage biopsy seeded scaffold	Collagen ↑, compressive modulus ↑, tensile modulus ↑	[85]
Heher et al. (2015)	Tension	Tension: 10% static strain for 6 hr, 3% static strain for 18 hr	6 days	Mouse myoblast C2C12 fibrin rings	MyoD ↑, Myogenin ↑, TnnT1 ↑	[82]
Scaglione et al. (2010)	Torsion	Torsion: 100° magnitude at a rate of 600°/min	3 days	3T3 fibroblast cell seeded scaffold	Collagen type I ↑, tenascin C ↑, collagen type III ↑	[89]
Chan et al. (2015)	Torsion	Torsion: ±2° magnitude, 1 Hz. Compression: 0.2 MPa magnitude, 1 Hz. Tested at 1, 4, and 8 hr/day	7 days	Bovine intervertebral disc explants	Viability ↓, total disc volume ↓, MMP13 ↓, glycosaminoglycans/hydroxyproline ratio ↑	[90]
Veronick et al. (2018)	Ultrasound	Ultrasound: 1 MHz carrier frequency pulsed at 1 kHz, 20% duty cycle, 30 and 150 mW/cm^2^	20 min	MC3T3‐E1 pre‐osteoblast seeded scaffold	Cyclooxygenase 2 ↑, prostaglandin E2 ↑	[92]

*Note*: Upregulation (↑), downregulation (↓), and no significant changes (−).

Single stimuli mechanical bioreactors use bone adaptation and mechanotransduction principles as a foundation to grow and maintain hard and soft tissues *in vitro*. The most commonly applied mechanical loads for these bioreactors are compression, tension, shear, torsion, and ultrasound. The type of mechanical stimulation, as well as the particular loading regime, defines the effectiveness of certain osteogenic responses, such as cell proliferation, differentiation, and viability.

Compressive forces are exhibited naturally on bone and cartilage *in vivo*. For example, they can be generated through direct axial loading or through the associated tensile and compressive forces from bending.^50^ Compressive loading can also generate pressure gradients that facilitate interstitial fluid flow to apply low shear strains on nearby osteocytes. Compression bioreactors can be used on either cell‐seeded scaffolds^11^, ^20^, ^78^ or tissue explants.^17^, ^38^, ^79^ Several groups have shown enhanced osteogenesis and chondrogenesis using compression bioreactors.^10^ For example, Hoffmann et al. and Tsai et al. reported increased extracellular matrix mineralization and deposition after dynamic loading.^80^, ^81^ Others have shown an increase in alkaline phosphatase (ALP), calcium ions, elastic moduli, proliferation, expression of osteogenic gene markers, cell viability, and vascularization.

In the field of orthopedics, tension bioreactors are typically used to facilitate the growth of muscles and tendons *in vitro*.^82^, ^83^, ^84^ However, they can also be used to grow some hard tissues, such as fibrocartilage, or more specifically the meniscus. The meniscus is subjected to both compressive and tensile forces *in vivo*. Thus, dynamic compressive and tensile forces have been applied to meniscus constructs in tandem with an observed increase in the following: proliferation, collagen, osteocalcin, and stiffness.^85^, ^86^

Unlike compressive and tensile forces, which are typically applied directly to the substrate, shear loading is applied to cells within bioreactors through controlled fluid flow. This fluid flow embodies the shear forces experienced by osteocytes and other bone cells *in vivo* due to blood flow and interstitial fluid flow.^50^, ^83^ Dynamic shear flow has been shown to increases both chondrogenesis and osteogenesis by applying physiological mechanical stimulation and enhancing mass transport, especially within the cores of the constructs.^21^, ^55^ There are three main patterns of fluid flow: continuous, pulsatile, and oscillatory. Most studies agree that pulsatile fluid flow is a much more potent stimulator of bone and cartilage cells compared with the other dynamic patterns.^3^ It is also important to note that if the rate of shear flow exceeds a certain threshold seeded cells may detach from the matrix. Thus, the optimal flow rate for each bioreactor setup should be determined based off the combination of cell types and scaffold material.^55^

Although the most common types of mechanical stimulation applied to bone and cartilage *in vitro* are compression, tension, and shear, recent literature has suggested that torsion also plays an important role in the mechanotransduction process, specifically for long bones.^87^ Torsion has been shown to promote the shaping of tubular structures in bone, which is essential for the development of shafts in long bones and the femoral neck.^88^ Thus far, torsion bioreactors have shown promising results, upregulating the gene expression of collagen type 1, tenascin C, and collagen type 3.^89^ Additional research is needed to determine the optimal loads and angles for torsional stimulation. In addition to long bones, torsion bioreactors are also used to culture intervertebral discs, since the spine is routinely subjected to compression and torsion.^87^, ^90^ With this dual loading in mind, some bioreactors are being designed with multiple levels of mechanical stimulation to better mimic *in vivo* conditions.

In the past few decades, ultrasound stimulation has been studied as a form of mechanical loading in bone and cartilage TE applications. Of particular interest is the effects of low‐intensity pulsed ultrasound (LIPUS) on bone healing properties.^91^, ^92^, ^93^ LIPUS is a gentle form of mechanical energy that is transmitted through tissue and is a widely used tool for therapy and diagnosis.^93^ LIPUS has been shown to affect the differentiation of osteoblasts and upregulates prostaglandin E_2_ and cyclooxygenase 2 for accelerated bone regeneration.^91^, ^92^, ^93^ Ultrasound has also been used in tandem with dynamic compressive loading, resulting in increased osteogenic gene expression compared with the individual stimuli.^20^

In summary, mechanotransduction can be triggered by any of the listed forms of mechanical stimulation. However, in order to push for clinical relevance, the loading types and regimes should be chosen to reflect the *in vivo* microenvironment of the specific tissue type. As such, the best *in vitro* microenvironment created by a bioreactor is likely one that incorporates several mechanical, as well as chemical and electrical, stimuli.

### 4.2 Electrical stimulation

Electrical stimulation has been studied as a method for enhancing bone healing for several decades. In the 1950s, Fukada and Yasuda discovered that the collagen molecules in bone exhibit piezoelectric properties, allowing the tissue to create a dipole moment when under strain.^52^ It was later discovered that streaming potentials, or electric fields generated by stress‐generated flow of ionic fluids, also exists in bone.^50^ Furthermore, when a bone is fractured, the gap within the fracture site becomes negatively charged, likely playing a role in attracting inflammatory and reparative cells.^94^ Since native bone tissue naturally exhibits these electrical properties, researchers have been looking at novel electrical stimulatory devices to promote bone regeneration and prevent nonunions during fracture repair or spinal fusion.

Commercially available electrical stimulation devices are commonly used in clinical applications to promote bone healing in fractures or other large bone defects. There are two variations of electrical stimulation devices: percutaneous (external) devices and transcutaneous (internal) devices.^95^ Percutaneous bone stimulators include capacitive coupling and inductive coupling devices, which generate electric and magnetic fields, respectively, to the defect site. Whereas a transcutaneous bone stimulator supplies direct current (DC) directly to the defect. Each of these methods can be adapted to be used in TE bioreactors to grow viable tissue constructs before being implanted into defects.

Studies have shown that electrical stimulation heavily influences cellular behavior, such as adhesion, migration, proliferation, differentiation, mineralization, extracellular matrix deposition, and vascularization, all of which are essential in BTE treatments (Table 3).^15^, ^18^, ^95^ However, as with mechanical stimulation bioreactors, the specific loading regime plays a vital role on the success of the treatment. For the percutaneous devices, the electrical stimulation is typically characterized by the electric field magnitude that permeates through the skin and soft tissue to interact with the bone defect site.^35^, ^96^, ^97^, ^98^, ^99^ On the other hand, transcutaneous DC devices report the electrical stimulation as a current, current density, charge, electric field, potential, or a combination of the previously mentioned.^18^, ^38^, ^97^, ^100^, ^101^, ^102^, ^103^ Recently, it has been speculated that the current density and electric field play a more important role in cell behavior mainly due to heating effects, which may explain the inconsistent results of studies that only reported on the current magnitude.^100^ Generally speaking, electric fields should remain below 10 V/cm and current density below 1–2 mA/cm^2^ to avoid damaging nearby tissue.^100^

**TABLE 3:** Summary of cellular behaviors in response to electrical loading for *in vitro* and animal studies

Articles	Loading type	Loading regime	Loading duration	Cell/tissue type or animal model	Results	References
Leppik et al. (2018)	Direct current	100 mV/mm, 1 hr/day	21 days	AT‐MSC seeded on ß‐TCP scaffold	Viability ↑, ALP ↑, TGF‐ß1 ↑, BMP2 ↑, osteopontin ↑, calmodulin ↑	[15]
Wang et al. (2016)	Direct current	200 mV/cm, 50% duty cycle, rectangular wave, 1–100 K Hz; 30 min/day	12 days	MC3T3‐E1 cells	For 100 Hz: Collagen type I ↑, collagen type 2 ↑, RUNX ↑, osteopontin –, proliferation ↑, calcium deposition ↑, ALP –	[101]
Cho et al. (2019)	Direct current	Constant: 100 μA pulsed: 100 μA, 100 Hz, 200 μs	56 days	Adipose tissue derived hMSC‐LCs seeded on nitinol mesh	Volume of fusion mass ↑, osteocalcin ↓, sclerostin ↑	[102]
Fredericks et al. (2007)	Direct current	100 μA constant DC (SpF‐100 device)	28 days	New Zealand white rabbit autologous bone grafts	BMP‐2 ↑, BMP‐6 ↑, BMP‐7 ↑, ALK‐2 –, ALK‐3 –, FGF‐2 –, TGF‐ß1 –, VEGF –	[32]
Zhang et al. (2013)	Direct current	200 μA constant DC 4 hr/day	21 days	MC3T3‐E1 osteoblasts	Metabolic activity ↑, ALP ↑, calcium deposition ↑, RUNX2 ↑, osterix ↑, osteopontin ↑, osteocalcin ↑	[103]
Clark et al. (2014)	Capacitively coupled	20 mV/cm, 60 kHz, 50% duty cycle, 2 hr/day	21 days	Human calvarial osteoblasts	ALP ↑, BMP‐2 ↑, BMP‐4 ↑, TGF‐ß1 ↑, TGF‐ß2 ↑, TGF‐ß3 ↑, FGF‐2 ↑, osteocalcin ↑	[98]
Krueger et al. (2019)	Capacitively coupled	Alternating voltage (RMS values): 100 mV (5.2 × 10^−5^ mV/cm) and 1 V (5.2 × 10^−4^ mV/cm) at 1 kHz; 45 min of stimulation 3 times/day	7 days	Osteoarthritic and non‐degenerative hyaline cartilage derived human chondrocytes seeded scaffold	For 100 mV: Collagen type I ↑, collagen type II ↑, GAG ↑	[35]
Gittens et al. (2013)	Electrical polarization without exogenous current	0 mV, +100 mV, −100 mV, −200 mV, −300 mV, −400 mV, −500 mV; 2 hr of stimulation, 22 hr of incubation	1 day	Plated MG63 cells	As potential decreases: proliferation ↓, osteocalcin ↑, osteoprotegerin ↑, VEGF ↑	[18]
Suryani et al. (2019)	Pulsed electromagnetic fields	4.40 ± 0.04 V at 50.00 ± 0.01 Hz and a pulse duration of 3.00 ms; stimulated for 0, 15, 30, and 60 min/day	28 days	Plated murine MC3T3‐E1 subclone 4 cells	Viability –, mineralization –, bone sialoprotein ↑ (30 min/day on day 7), osteocalcin –	[96]
Chang et al. (2004)	Pulsed electromagnetic fields	2 mV/cm, 15 Hz, 0.1 mT, 8 hr/day	14 days	Neonatal mouse calvarial bone cell	ALP ↓, proliferation ↑, ECM synthesis –, osteoprotegerin ↑, RANKL ↓	[99]

*Note*: Upregulation (↑), downregulation (↓), and no significant changes (−).

Although electrical stimulation in its various forms has been extensively used in clinical and BTE applications, the specific mechanisms of action are not fully understood. However, a few hypothetical mechanisms have been proposed and are actively being studied.^43^, ^44^ For example, sclerostin is known to play a significant role in the bone healing process by inhibiting the Wnt pathway. Detailed studies on the structure of sclerostin have identified binding sites for heparin, a highly negatively charged molecule that plays a role in localizing sclerostin to the surface target cells.^104^ Some of the possible effects electrical stimulation could have on sclerostin include the denaturation of the molecule preventing heparin binding, disrupting the electrical interaction between sclerostin and heparin, or introducing negative ions that bind to sclerostin and block heparin. Several other signal transduction pathways have been suggested as a potential mechanism of action for electrical stimulation but for any of these hypothetical mechanisms to be considered proven, more extensive research with standardized testing procedures needs to be conducted.

### 4.3 Chemical stimulation

Chemical stimulation controls both spatial and temporal factors which causes various types of changes, such as a metabolic or phenotypic alteration in the cells of interest. Within TE, chemical stimulation means the addition of key small molecules, peptides, or proteins to promote physiological interactions on a molecular level. Specific to orthopedics, this involves nutrients, growth factors, cytokines, and hormones, for the initiation, promotion, or inhibition of hard or soft skeletal tissue regeneration. One example of chemical stimulation in TE is the use of recombinant human bone morphogenetic proteins (rhBMP) to increase cell differentiation into osteoblasts.^105^ Knowledge of these molecules and their interaction with skeletal tissues, specifically bone, can be further studied within a biomimetic bioreactor.

There has been extensive study into the various types of chemical stimulation factors. Specifically, how concentration, frequency, and interaction with supplementary molecules change the outcome of the addition, deficit, or therapeutic effects caused by these factors. There are extensive reviews and studies of these results for proteins such as the transforming growth factor‐β (TGF‐β),^106^, ^107^, ^108^, ^109^ bone morphogenetic proteins (BMPs),^108^, ^110^, ^111^ (specifically, BMP‐2^43^, ^76^, ^112^, ^113^ though BMP‐2, BMP‐4, BMP‐5, BMP‐6, BMP‐7, and BMP‐9 have been known to initiate signaling cascades for bone formation^76^), Small Mothers Against Decapentaplegic (SMAD),^114^ and vascular endothelial growth factor (VEGF).^115^, ^116^ Additionally, hormones associated with orthopedics like estrogen,^117^, ^118^ androgen,^119^, ^120^ and parathyroid hormone (PTH)^121^, ^122^, ^123^ have been studied immensely. Other important molecules found in orthopedics are calcium,^124^ vitamins (specifically vitamin D^124^, ^125^), and nitric oxide (NO).^126^, ^127^, ^128^

The small molecules involved in orthopedics make up key aspects of the bone regeneration pathways.^43^ This led to increased interest in determining how each of the various bone regeneration pathways operate. The widespread investigation into the various signaling pathways involved in the bone regeneration such as the Wnt/β‐catenin,^129^, ^130^, ^131^, ^132^, ^133^ Notch,^134^, ^135^, ^136^ BMP/TGF‐β,^137^, ^138^ Pi3K/Akt/mTOR,^139^ and mitogen‐activated protein kinase (MAPK).^140^

Instead of focusing on the individual effects of various chemical stimuli on mechanotransduction and bone healing processes, this review draws attention to the combinatorial effects (mechanical + chemical and electrical + chemical) which provide a more clinically relevant environment for improved patient care. While elucidating the role of each individual signaling molecule is vital for understanding development and disease, combining these factors to mimic the physiological environment will provide a substantially more clinically relevant condition (Table 4). While this is not an extensive list of all the chemical stimuli that could be implemented in a biomimetic bioreactor, it represents the concept of chemical agents utilized to mimic *in vivo* situations.

**TABLE 4:** Summary of cellular behaviors in response to a combination of chemical and mechanical or electrical loading for *in vitro* and animal studies

Articles	Loading type	Loading regime	Loading duration	Cell/tissue type or animal model	Results	References
Kim et al. (2003)	Parathyroid hormone and compression	15 μg/kg/day rat PTH (1–34); 0, 50, and 100 N uniaxial compressive loading, 1 Hz, 300 cycles/day	28 days	Male Sprague–Dawley rats	For PTH + 100 N: Bone formation rate ↑, mineral apposition rate ↑, labeled bone surface ↑	{71]
Carvalho et al. (1994)	Parathyroid hormone and tension	20 kPa (<1% strain) at 0.05 Hz, 10 s strain and 10 s relaxation	0.5, 1, 5, 10, and 30 min; 1, 3, and 7 days	Bone cells from Sprague–Dawley rats	IP3 ↑, PKC activity ↑, cAMP ↑	[144]
Chow et al. (1998)	Parathyroid hormone and compression	6 or 60 μg/kg human PTH (1–34); 150 N (700 μstrain), 1 Hz, 30 cycles	30 s	13‐week‐old female Wistar rats	Bone formation rate ↑, mineral apposition rate ↑, labeled bone surface ↑ (compression had greater compared with PTH)	[145]
Ryder et al. (2000)	Parathyroid hormone and bending/fluid shear	50 nM bPTH (1–34); 4,230 μstrain, 0.2 mm/s and 3 mm/s, four‐point bending; 12 and 25 dynes/cm^2^	30 min (bending) and 3 min (fluid shear)	MC3T3‐E1 cells	For PTH and bending: COX‐2 mRNA levels ↑; for PTH and fluid shear: calcium levels ↑	[146]
Jagger et al. (1996)	Estrogen and compression	40 mg/kg estradiol; 150 N (700 μstrain), 1 Hz, 300 cycles	5 min	13‐week‐old female Wistar rats	E2 + compression compared with compression: Bone formation rate ↓, mineral apposition rate –, labeled bone surface ↓	[147]
Allison et al. (2019)	Estrogen and perfusion	Estrogen (10 nM 17β‐estradiol), selective estrogen receptor degrader (10 nM 17β‐estradiol + 100 nM fulvestrant), and estrogen withdrawal after 3 days; 9.2 ml/min, 0.5 Hz	1 hr	Murine monocyte/macrophage RAW264.7 cells and MC3T3‐E1 osteoblast‐like cells	For estrogen deficiency: cyclooxygenase‐2 ↑, macrophage colony‐stimulating factor ↑, osteoprotegerin –	[148]
Neumann et al. (2015)	BMP‐2 and compression	A first‐generation, E1‐deleted, E3‐deleted, serotype 5 adenoviral vectors carrying the cDNA for human BMP‐2 (Ad.BMP‐2); 10% strain, 1 Hz, 1 hr/day, 6 days/week	7 and 28 days	hACPCs seeded onto polyurethane scaffold	RUNX2 –, SOX9 –, Aggrecan ↑, collagen type I –, collagen X –, ALP ↑, GAG ↑	[149]
Kopf et al. (2012)	BMP‐2 and compression	5 nM BMP‐2; 10% strain, 1 Hz	120 min and 24 hr	hFOB 1.19 cell seeded on collagen scaffold	BMP‐2 –, BMP‐4 ↓, BMP‐6 ↑, BMP‐7 ↓, Noggin ↑, Id1 ↑, Id2 ↑, c‐fos ↑, RUNX2 ↓, osteopontin ↑, Dlx5 –, Dlx2 ↑, Dlx3 ↑	[150]
Zhang et al. (2013)	BMP‐2 and direct current	50 μl of BMP‐2 (0.2, 1, 5 μg/ml); 4 hr/day, 200 μA	7 days	Osteoblasts seeded on polypyrrole/chitosan films	Metabolic activity ↑, ALP ↑, calcium deposition ↑	[103]
Wang et al. (2017)	BMP‐7 and capacitively coupled	Noggin (400 and 600 ng/ml), BMP‐7 (10, 20, and 50 ng/ml); 17.33 mV/cm, 60 kHz, 4 hr/day	7 days	Human disc nucleus pulposus cells	Aggrecan ↑, collagen type II ↑	[151]

*Note*: Upregulation (↑), downregulation (↓), and no significant changes (−).

## 5 MULTIPLE STIMULI BIOREACTORS

There has been a gradual progression in TE from single source stimuli, such as one type of mechanical loading, electrical loading, or chemical stimulation, to more complex systems that incorporate a combination of multiple stimulation types. These complex systems can manifest themselves in the form of integrated mechanical loads, such as cyclic compression and shear stress, to try to represent the multiple forces present in bones physiologically.^141^ On the other hand, combinations across stimulation types are also possible, such as integrating the chemical factor PTH with mechanical compression.^71^, ^141^ While many groups have attempted to combine multiple stimulations in an experimental environment, fewer have integrated these designs into functioning bioreactors. In the following sections, examples of some of these multiple stimulation bioreactors are provided, which include mechanical + mechanical,^20^, ^141^, ^142^, ^143^ mechanical + chemical,^71^, ^144^, ^145^, ^146^, ^147^, ^148^, ^149^, ^150^ electrical + chemical,^103^, ^151^ and mechanical + electrical combinations.^46^

Common combinations of mechanical stimuli include, but are not limited to, compression and shear,^141^, ^142^ compression and ultrasound,^20^ and compression and torsion.^143^ As mentioned previously, shear forces are often integrated into multiple stimulation bioreactors via fluid flow. However, some groups, such as Vainieri et al., have used direct shear forces with compression to simulate ball and socket motion associated with articulating joints. Vainieri et al. designed a cartilage bioreactor that provided a multiaxial motion to get closer to the natural physiological joint kinematics, so that they could study cartilage defects in bovine stifle joints.^141^ This bioreactor was designed to address problems where articular cartilage lesions present poor healing capacities while also providing a suitable environment for biomaterials and implants to be assessed.^141^ The results of this study showed no detrimental effects from the dual mechanical loading approach on the cartilage samples and an upregulation in proteoglycan‐4 (*PRG4*) gene expression, which is responsible for joint lubrication.^141^ Despite the success of the study, Vainieri et al. noted that additional adaptations to this cartilage bioreactor, such as cartilage‐on‐cartilage articulating motion, the addition of cytokines, and the addition of factors that affect the immune system's ability to respond to injuries to the synovium would aid in recapitulating the *in vivo* environment.^141^, ^152^

The combination of chemical and mechanical factors in a contained environment has been shown to aid in bone healing. For example, the synergistic effects of PTH with compression was exemplified in a study by Kim et al.^71^ The results of this study showed that the combination of both stimuli significantly increased the bone formation rate compared with the diminishing effects of only one of the two stimuli.^71^ Single stimuli showed initial positive results, however, after 4 weeks the bone response diminished to the level of baseline control animals.^71^ Another group analyzed the phenotypic differences in calvarial and femoral osteoblastic responses to several chemical and mechanical factors, including the induction of osteogenesis through compressive loading, estrogen, growth factors, and cytokine stimulation.^153^ The goal of this study was to investigate if there was a difference response in these two types of bone cells since skull/calvarial bones tend to have osteoporosis‐resistant nature.^153^ The mechanical loading results of this study showed that there was an induction of two early response genes expression in femoral osteoblasts but remained unchanged in the calvarial osteoblasts.^153^ Additionally, the estrogen receptor beta (ERβ) expression was upregulated in calvarial osteoblasts, and the estrogen responsive transcriptional repressor (RERG) was expressed 1,000‐fold greater levels in calvarial osteoblasts compared with femoral osteoblasts.^153^ Ultimately, the results of the study showed how there are functional differences between calvarial and femoral osteoblasts *in vivo* and a better understanding might lead to a better therapeutic prospect.^153^

The pilot ovine spinal interbody device study conducted by Friis and Arnold involved a mixed mode of mechanical and electrical stimulation.^46^ To investigate the pathway triggered in response to the dual modes of stimuli there is a need for a biomimetic bioreactor to quantify on a cellular level the physiologic mechanism of this increased bone growth. Coined mechanically synced electrical stimulation (MSES), the synergistic effects of physiological loading of the sheep to initiate the piezoelectric circuit in the interbody device showed better bone healing in less time than the controls.^46^ Knowing what pathway is utilized due to the introduction of the interbody device and physiological mechanical loads would allow for clinically applicable and translatable information.

**FIGURE 7:**
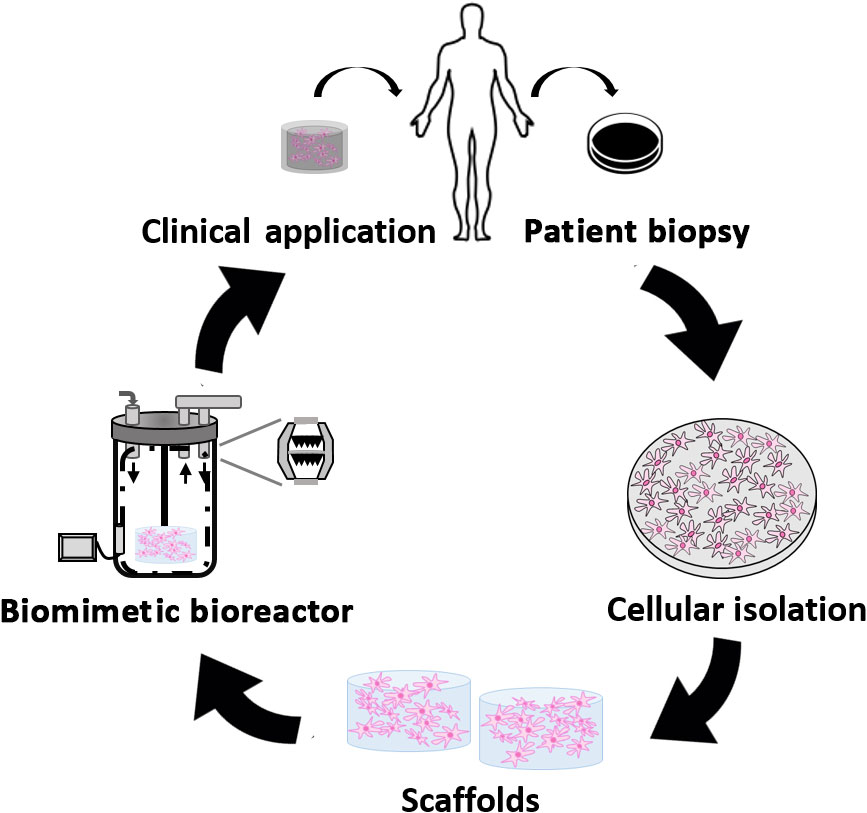
Flowchart illustrating the practical application of a clinically relevant biomimetic bioreactor for specific patient needs

Progress toward clinically relevant bioreactors has been made, but a true physiological representation of cell microenvironments has yet to be realized. However, recent studies are reaffirming the notion that physiological loads are critical for enhancing cellular behaviors. One approach could be mimicking the physiological responses, with all forms of stimuli, such that the environment promotes innate cell and tissue repair processes during disease or injury. It would be interesting, and of clinical relevance, to design a system where such responses could be controlled by variable inputs integrated within a reactor system (Figure 7).

## 6 FUTURE DIRECTIONS

Diversification of bioreactors enables researchers to better understand how various changes in physiologic conditions affect the human body. The human body is an extremely complicated machine that makes the translation between benchtop to bedside particularly difficult. Designing a clinically relevant biomimetic bioreactor that simultaneously affects mechanical, electrical, and chemical stimuli would further our understanding and reduce time on a design that would not work in clinical practice. While there are a few ways to interact with a bioreactor system, batch, fed‐batch, and continuous batch systems, we believe that continuous batch systems will better recapitulate and mimic the physiological environment. Additionally, it will take a bioreactor that can provide mechanical stimulation, apply electrical loads, and incorporate chemical factors to fully see the multiple stimulation machine come together. Studying how each one of these cues and factors are incorporated into the system would lead to better engineered orthopedic devices.

## CONFLICT OF INTEREST

The authors declare no conflicts of interest.

## Data Availability

Data sharing is not applicable to this article as no new data were created or analyzed in this study.
